# *Bacillus subtilis* Attenuates Hepatic and Intestinal Injuries and Modulates Gut Microbiota and Gene Expression Profiles in Mice Infected with *Schistosoma japonicum*

**DOI:** 10.3389/fcell.2021.766205

**Published:** 2021-11-16

**Authors:** Datao Lin, Qiuyue Song, Yishu Zhang, Jiahua Liu, Fang Chen, Shuling Du, Suoyu Xiang, Lifu Wang, Xiaoying Wu, Xi Sun

**Affiliations:** ^1^ Department of Parasitology, Zhongshan School of Medicine, Sun Yat-Sen University, Guangzhou, China; ^2^ Key Laboratory of Tropical Disease Control, Ministry of Education, Guangzhou, China; ^3^ Provincial Engineering Technology Research Center for Diseases-vectors Control, Guangzhou, China; ^4^ Department of Clinical Laboratory, Xiangyang No.1 People’s Hospital, Hubei University of Medicine, Xiangyang, China; ^5^ School of Medicine, South China University of Technology, Guangzhou, China; ^6^ The Third Affiliated Hospital, Sun Yat-sen University, Guangzhou, China

**Keywords:** probiotics, *Schistosoma japonicum*, pathological injury, gut microbiota, transcriptomics

## Abstract

Parasitic infection can induce pathological injuries and impact the gut microbiota diversity and composition of the host. *Bacillus subtilis* is a nonpathogenic and noninvasive probiotic bacterium for humans and other animals, playing an important role in improving the host immune system’s ability to respond to intestinal and liver diseases and modulating gut microbiota. However, whether *B. subtilis* can impact biological functions in *Schistosoma japonicum*–infected mice is unclear. This study used oral administration (OA) of *B. subtilis* to treat mice infected with *S. japonicum*. We evaluated changes in the gut microbiota of infected mice using 16 S rRNA gene sequencing and differentially expressed gene profiles using transcriptome sequencing after OA *B. subtilis*. We found that OA *B. subtilis* significantly attenuated hepatic and intestinal pathological injuries in infected mice. The gut microbiota of mice were significantly altered after *S. japonicum* infection, while OA *B. subtilis* remodel the diversity and composition of gut microbiomes of infected mice. We found that the *S. japonicum–*infected mice with OA *B. subtilis* had an overabundance of the most prevalent bacterial genera, including *Bacteroides*, *Enterococcus*, *Lactobacillus*, *Blautia*, *Lachnoclostridium*, *Ruminiclostridium*, and *Enterobacter.* Transcriptomic analysis of intestinal tissues revealed that OA *B. subtilis* shaped the intestinal microenvironment of the host responding to *S. japonicum* infection. Differentially expressed genes were classified into KEGG pathways between *S. japonicum–*infected mice and those without included cell adhesion molecules, intestinal immune network for IgA production, hematopoietic cell lineage, Fc epsilon RI signaling pathway, Th1 and Th2 cell differentiation, Th17 cell differentiation, calcium signaling pathway, Fc gamma R-mediated phagocytosis, chemokine signaling pathway, phospholipase D signaling pathway, NF-kappa B signaling pathway, B cell receptor signaling pathway, pancreatic secretion, and phagosome. In conclusion, our findings showed that OA *B. subtilis* alleviates pathological injuries and regulates gene expression, implying that *B. subtilis* supplementation may be a potential therapeutic strategy for schistosomiasis. Our study may highlight the value of probiotics as a beneficial supplementary therapy during human schistosomiasis, but further studies are needed.

## Background

Schistosomiasis, a result of infection with blood flukes of *Schistosoma*, is considered as a neglected tropical disease, causing over 250 million people to be infected globally and threatening nearly one-eighth of the world population (Colley et al., 2014; McManus et al., 2018). This zoonotic disease, which seriously damages human and animal health and hinders socioeconomic development, can be found in more than 78 countries in South America, Asia, and Africa (Lin et al., 2020; McManus et al., 2018). In China, *Schistosoma japonicum* is the only endemic parasitic flatworm of schistosomes and is mainly prevalent in 12 provinces along the middle and the lower reaches of the Yangtze River and in southern regions. *S. japonicum* infection remains one of the most important public health problems in mainland China, and there are still more than 29,214 advanced schistosomiasis cases documented in 2019 (Zhang LJ. et al., 2020).

*S. japonicum* infection can cause diarrhea, fatigue, and anemia in the early stages and cause portal vein hypertension syndrome, ascites, and hepatic fibrosis in the later stages. Previous studies have demonstrated that schistosomal eggs, not adult worms, are the key factor inducing hepatic fibrosis and even morbidity (Barnett, 2018; Colley et al., 2014; McManus et al., 2018). The mature schistosome lays a large number of eggs in the vessels of the intestinal wall during schistosomiasis progression (Pearce and MacDonald, 2002). Many laid eggs are deposited in the liver via the portal system and finally induce portal vein hypertension syndrome, ascites, granuloma formation, and hepatic fibrosis (Colley et al., 2014; McManus et al., 2018; Pearce and MacDonald, 2002). Evidence suggests that upregulation of the serum cytokine levels of interleukin 13 (IL-13), IL-5, IL-4, and TGF-β may lead to hepatic fibrosis (Hu et al., 2020; Qiu et al., 2017). Studies have indicated that deposited eggs stimulate dominant CD4^+^ Th2 immune responses accompanied by eosinophil, macrophage, hepatic stellate cell, and lymphocyte recruitment and then induce granuloma formation (Burke et al., 2009; Colley and Secor, 2014). Shifts in the Th1 response to the Th2 response are a determining factor in the mechanism of granuloma formation and hepatic fibrosis (Bourke et al., 2013; Qiu et al., 2017; Romano et al., 2016). Targeting the Th1/Th2 balance in *S. japonicum*–infected mice can attenuate hepatic fibrosis (Huang et al., 2020). Furthermore, a schistosome infection can also cause a wide range of clinical symptoms such as gut inflammation and affect the gut microbiota of mice (Hu et al., 2020). However, the mechanism of the complex interaction between host immunity and *S. japonicum* remains unclear.

Mammals harbor diverse bacteria that affect host biology and health in various ways. Previous studies have shown alterations in composition and structure of the gut microbiome and metabolite profiling in *S. japonicum*–infected mice (Hu et al., 2020; Song et al., 2020; Zhang B. et al., 2020). Evidence suggests that gut microbiota composition is associated with *Schistosoma mansoni* infection burden in rodent models (Cortes et al., 2020). Our previous work and other reports revealed that schistosome infection decreases the alpha diversity and richness of beneficial bacteria of gut microbiota in mammals such as rodents (Anter et al., 2020; Cortes et al., 2020; Hu et al., 2020; Jenkins et al., 2018; Song et al., 2020; Zhang B. et al., 2020) and humans (Gui et al., 2021; Kay et al., 2015). Lack of host gut microbiota alters host immune responses to intestinal granuloma formation and hepatic fibrosis in infected mice (Holzscheiter et al., 2014). Therefore, a feature is that the gut microbiota of hosts during schistosomiasis is lost and lacking. Homeostasis of the composition and diversity of gut microbiota can be beneficial for host health and biological functions after schistosome infection.

*Bacillus subtilis*, a member of class *Bacilli* significantly increased in humans after schistosome infection (Gui et al., 2021), is a nonpathogenic and noninvasive probiotic bacterium for humans and other animals. *B. subtilis* is one of the 42 probiotics that can be orally administered directly and was announced by the Food and Drug Administration (FDA) in 1989. Evidence suggests that oral administration (OA) of *B. subtilis* protects HFD-induced obese mice against obesity and modulates host gut microbiota (Huang et al., 2021; Lei et al., 2015). *B. subtilis* inhibits the occurrence of ulcerative colitis via changes in the intestinal microecology (Wu et al., 2019). *B. subtilis* produces surfactin, an antibacterial peptide, which protects the host against copper sulfate–induced inflammation and hepatic injury in zebrafish (Wang et al., 2021). In short, *B. subtilis* plays an essential role in improving the host immune system’s ability to respond to intestinal and liver diseases. Evidence has also suggested that *Bacillus* species could inhibit the growth of intestinal pathogens such as *Escherichia coli*, *Helicobacter pylori*, *Staphylococcus aureus*, and *Clostridium difficile* (Duc et al., 2004; Colenutt and Cutting, 2014; Piewngam et al., 2018)*.* However, whether *B. subtilis* can impact intestinal and hepatic pathological injuries in mice infected with *S. japonicum* is unclear.

In this study, we hypothesized that *B. subtilis* would affect intestinal and hepatic injuries in *S. japonicum*–infected mice and modulate the biological aspects of hosts. To explore this hypothesis, we collected hepatic, intestinal, and stool samples from *S. japonicum*–infected mice treated with or without OA *B. subtilis*. Subsequently, we evaluated pathological progression via histological detection, investigated alterations in the gut microbiota via 16 S rRNA gene sequencing, and analyzed the gene expression profiles using transcriptomics through collected samples. Our study demonstrated that OA *B. subtilis* may be a beneficial supplementary therapy for human schistosomiasis.

## Methods

### Ethics Approval and Consent to Participate

All experiments were conducted in strict accordance with the Guide for the Care and Use of Laboratory Animals of the National Institutes of Health. The animal experiments were reviewed and approved by the Institutional Animal Care and Use Committee of Sun Yat-sen University (Permit No: 2016-104) and the Medical Research Ethics Committee of Sun Yat-sen University (SYSU-IACUC-2019-B517).

### Preparation of Probiotic Bacterial Strain

*B. subtilis* CMCC(B) 63501 was purchased from Solarbio Science and Technology Co., Ltd (Beijing). *B. subtilis* was cultured in a lysogeny broth (LB) medium to spawn for 24 h. Then, bacteria were successively washed with 1 M NaCl and 1 M KCl and washed two times with distilled water. The LB agar plates were incubated at 27°C for 12–24 h, and the number of CFUs per plate was counted. Finally, the concentration of bacterial suspension was adjusted to 3 × 10^8^ CFU/ ml, 3 × 10^9^ CFU/ ml, or 3 × 10^10^ CFU/ ml. Each animal was given 0.3 ml of the final suspension every 3 days. The bacteria suspension was refreshed every week.

### Mice, Cercariae, Infection, and Probiotic Treatment

A total of 36 male pathogen-free BALB/c mice, approximately 6 weeks old (body weight: 18 ± 2 g), were purchased from the Experimental Animal Center of Southern Medical University. They were reared in plastic cages with free access to autoclaved chow and water in the Biosafety Level-2 (BSL-2) laboratory of Sun Yat-sen University under controlled temperature and humidity and a 12-h light and 12-h dark cycle. The animals were randomly divided into groups for further experiments. *Oncomelania hupehensis* was purchased from the Chinese Center for Disease Control and Prevention (Shanghai). After acclimating to the laboratory environment, each mouse was infected with 20 ± 2 *S. japonicum* cercariae via shaved abdominal skin. The probiotic supplement for mice was performed 1 week before the first day of infection, and treatment continued for 7 weeks. To conduct experiments, we randomly divided mice into four groups: normal group (NG), normal mice with OA *B. subtilis* group (NBS), *S. japonicum–*infected group (SI), and *S. japonicum–*infected mice with OA *B. subtilis* group (SIBS).

### Sample Collection

The mice were sacrificed after chloral hydrate asphyxiation and cervical dislocation of *S. japonicum* at 56 days postinfection (dpi). Left liver lobes and colons were collected and immediately fixed in 4% paraformaldehyde for histopathological analysis. Liver samples were also collected for hydroxyproline content measurement according to the protocol of the hydroxyproline assay kit (Nanjing, China). Stool samples were collected the day before sacrifice. Blood samples were drawn from orbital veins and centrifuged at 1,500 × *g* for 15 min, and then the serum was collected after clotting. In addition, both male and female *S. japonicum* worms were collected from the portal vein. The detections of worm length, worm burden, and egg burden were determined as described in a previous study (Shen et al., 2017).

### Histological Staining

For histopathological analysis, fixed fragments from the intestine and liver of mice were sliced into sections (5 μm thick). These slices were subjected to H&E staining and Masson’s trichrome staining. Images were captured under an inverted microscope (Olympus, Japan). The percentage of the fibrotic area was detected using a ZEISS Axio Scan. Z1 automated slide scanner microscope (Germany). A full view of the whole tissue was also obtained. We analyzed the whole tissue area and the blue-positive region using Image-Pro Plus 6.0 software (Media Cybernetics, USA). We calculated the percentage of the fibrotic area based on the area of the blue-labeled region/the total area of the whole liver tissue. Granulomatous responses in the colon were estimated by calculating the ratio of the integrated optical density (IOD) to the intestinal tissue area.

### RNA Extraction, RNA Sequencing, and Sequence Analysis

The liver and intestinal tissues were collected and stored in TRIzol reagent (Invitrogen, USA) at −80°C until processing. Total RNA was extracted as described in a previous study (Lin et al., 2020). We quantified the total RNA using a NanoDrop 2000 spectrophotometer (Thermo Scientific, America).

RNA integrity was assessed using the RNA Nano 6000 Assay Kit protocol in the Agilent Bioanalyzer 2100 system (USA). Total RNA was used as input material for the RNA sample preparations. PCR was performed with Phusion high-fidelity DNA polymerase, universal PCR primers, and index (X) primers. The PCR products were purified. The library quality was assessed on a Qubit2.0 Fluorometer and Agilent Bioanalyzer 2100 system. The prepared library was sequenced on an Illumina NovaSeq platform by Frasergen Company (Wuhan, China) and 150 bp paired-end reads were generated. After quality control of raw data, we carried out transcriptome assembly, gene functional annotation, differential expression analysis, KEGG enrichment analysis, and GO enrichment analysis. Genes with an adjusted fold change ≥2 were assigned as DEGs using the DESeq2 R package (1.20.0). The cluster profiler R package was used to test the statistical enrichment of DEGs in KEGG pathways.

### DNA Extraction

All stool samples were frozen at −80°C for further study. Total DNA from stool samples was isolated under a sterile environment according to the Hipure Stool DNA Kit protocol (Magen, China). After extraction, the total DNA quality and quantity examination were conducted using a NanoDrop 2000 spectrophotometer (Thermo Scientific, America).

### PCR Amplification and Sequencing

The V3–V4 region of the 16 S rRNA gene (approximately 500 bp) was amplified using a specific bacterial primer set (forward primer 5′-ACT​CCT​ACG​GGA​GGC​AGC​A-3′ and reverse primer 5′-GGACTACHVGGGTWTCTAAT-3′) and sequenced on an Illumina HiSeq 2500 platform. PCR amplification was performed using Takara PrimeStar DNA polymerase (China). The following PCR cycling conditions were used: denaturation at 95°C for 5 min, 25 cycles of 95°C for 30 s, 50°C for 30 s, 72°C for 40 s, and final extension at 72°C for 5 min. The PCR products were analyzed by 2% agarose gel electrophoresis. Finally, sequencing was performed by Biomarker Technologies (China).

### Analysis of Sequencing Data

After the base calling analysis, the raw data files from the sequencing platform were transformed into the original sequenced reads and stored in FASTQ format. QIIME (version 1.8.0) was used to cluster reads into operational taxonomic units (OTUs) and identified at 97% or more similarity (Caporaso et al., 2010). We rarified the OTU table and calculated the species abundance based on the OTUs and ACE indices using mothur (version 1.33.3) (Grice et al., 2009). To analyze the alpha diversity. As measures of beta diversity of the similarity, the nonmetric multidimensional scaling (NMDS) and partial least squares discrimination analysis (PLS-DA) plots with binary Jaccard distance were performed in R with the vegan package (Kambura et al., 2016). PERMANOVA was used to evaluate the beta diversity between samples or groups using mothur (version 1.33.3). We computed and explored the taxonomic content of the sequencing dataset using MEGAN (Huson et al., 2007). A ternary plot of the microbial community was generated as described in a previous study (Han et al., 2018). Based on the genus abundance of the gut microbiota of mice, random forest algorithms with 1,000 random permutations were conducted (Li et al., 2021). For range adjustment, all pairwise comparisons between two groups were tested using Student’s *t*-test.

### Statistical Analysis

We calculated the results using GraphPad Prism version 6.0 (USA). Data are expressed as the mean ± SEM. The differences between groups were analyzed by Student’s *t*-test using SPSS 19.0 software (USA). ^*^ *p* < 0.05, ^**^ *p* < 0.01, and ^***^ *p* < 0.001 were considered statistically significant.

## Results

### Protection of Mice Against Schistosome Infection by OA *B. subtilis*

**FIGURE 1:**
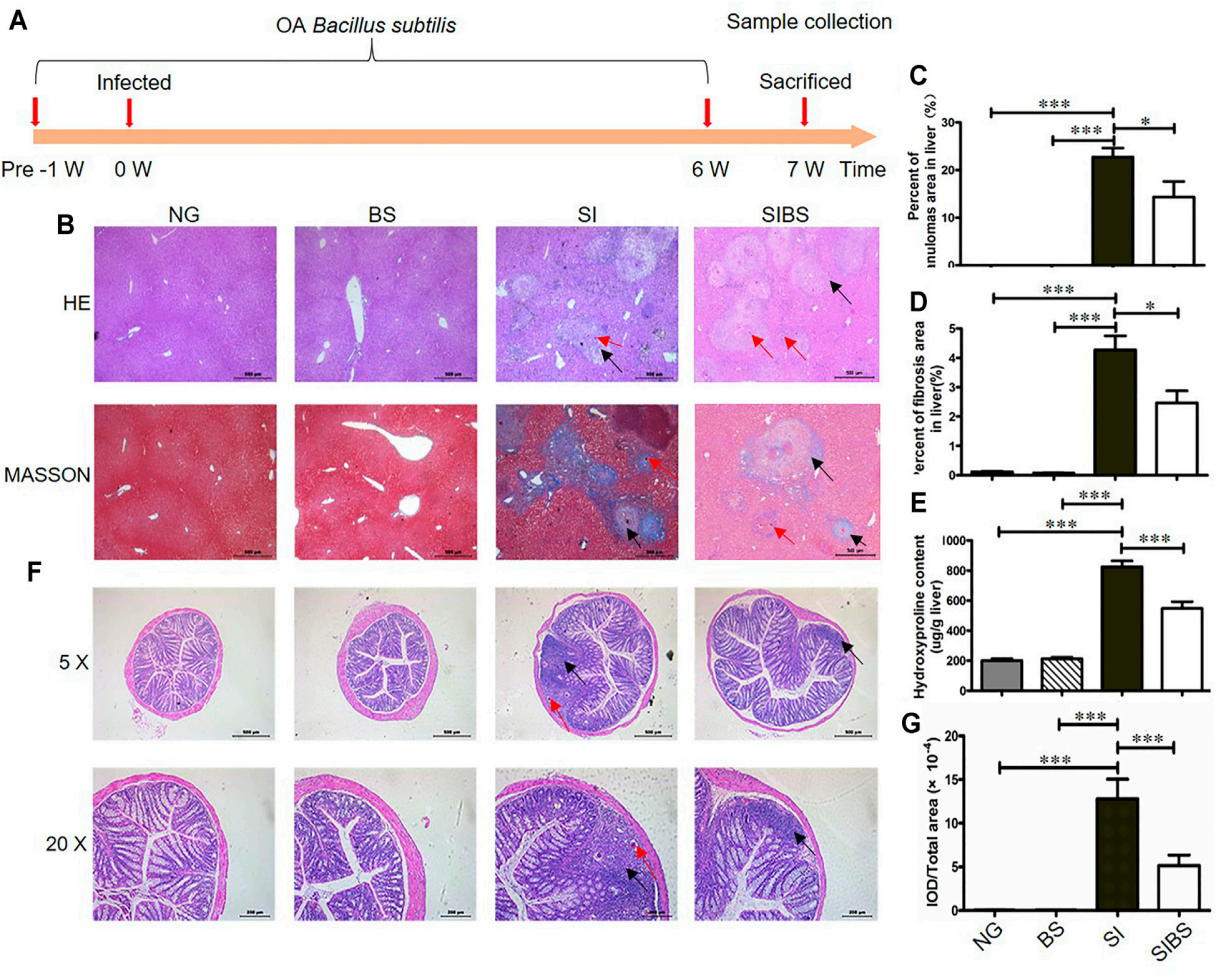
OA *B. subtilis* attenuated *S. japonicum*–induced hepatic and intestinal granulomas in mice (*n* = 6). **(A)** Time schedule for parasite infection, OA *B. subtilis*, and sample collection. Mice were infected with 20 ± 2 *S. japonicum* cercariae at 0 weeks. Infected mice received *B. subtilis* at a dose of 0.3 × 8 × 10^8^ CFU/ ml or PBS at 1 week post-infection. Samples were collected at the indicated time points. **(B)** H&E staining and Masson’s trichrome staining of liver tissues. **(C)** The value of granuloma area in the liver. **(D)** The value of fibrosis area in the liver. **(E)** Hydroxyproline content in the liver. **(F)** H&E staining of colon tissues. **(G)** The value of granuloma area in the colon. Granulomatous responses in the colon were estimated by calculating the ratio of the integrated optical density (IOD) to the intestinal tissue area. NG: normal mice with PBS group. BS: normal mice with OA *B. subtilis* group. SI: *S. japonicum–*infected mice group. SIBS: *S. japonicum–*infected mice with OA *B. subtilis* group. Black arrows indicate granulomas or fibroses and red arrows indicate schistosome eggs. * and *** were considered statistically significant.

To examine the role of *B. subtilis* in schistosomiasis-related hepatic fibrosis *in vivo*, we infected the mice with a lethal dose of *S. japonicum* cercariae. The infected mice were gavaged with *B. subtilis* or PBS every 3 days. Experimental designs on schedule for parasite infection, OA *B. subtilis* or PBS, and sample withdrawal are shown (Figure 1A). The survival rate of mice is shown (Supplementary Figure S1A).

Hepatic pathological injuries and fibrosis are severe symptoms during schistosomiasis and may cause death in hosts. Therefore, we next investigated whether OA *B. subtilis* (3 × 10^8^ CFU/ ml) protection involved attenuation of hepatic injuries and fibrosis. Based on the results of Masson’s trichrome staining (Figures 1B–D) and hydroxyproline quantification (Figure 1E), we found that infected mice displayed a significant reduction in the total area of hepatic granulomas and fibrosis after OA *B. subtilis*. In addition, the worm burden, egg count in the liver, and intestine and worm length from the mice were not significantly different between the groups (Supplementary Figures S1B–D).

However, whether the dead *B. subtilis* or living *B. subtilis* plays a role in attenuating pathological injuries in *S. japonicum*–infected mice is unclear. Subsequently, the infected mice were administered living *B. subtilis* (SIBS) or heat-killed *B. subtilis* (SIDBS). We found no significant difference in the area of fibrosis between infected mice and infected mice with OA-dead *B. subtilis* (Supplementary Figures S2A, B), in addition to the differences in the hydroxyproline quantification (Supplementary Figures S2A, C). However, it was significantly different in infected mice with and without OA living *B. subtilis* (Supplementary Figures S2). These findings indicated that OA living *B. subtilis* attenuates hepatic pathological injuries in *S. japonicum*–infected mice.

In addition, schistosome infection causes a wide range of clinical symptoms including gut inflammation, mainly induced by releasing eggs trapped in the intestinal wall. We next investigated whether OA *B. subtilis* protection involved attenuation of intestinal injuries. We found that *S. japonicum* eggs were distributed in the submucosa or even involved in the mucosa of infected mice (Figure 1F). These results indicated that parasitic eggs may affect the integrity of the intestinal tissue. Importantly, we found that infected mice treated with OA *B. subtilis* displayed a significant reduction in the total area of intestinal granulomas, as shown by HE staining (Figures 1F,G).

### Modulating the Diversity and Community Structure of the Gut Microbiota in *S. japonicum*–Infected Mice After OA *B. subtilis*

Evidence suggests that *S. japonicum* infection contributes to the dysbiosis of gut microbiota in mice, so we next investigated whether OA *B. subtilis* can affect the gut microbiota of mice during schistosomiasis using 16 S rRNA gene high-throughput sequencing. We analyzed the bacterial community structure and diversity of the intestinal contents of 36 individual mice. A total of 2,813,530 valid reads and 2,515,622 clean tags were retained from 36 stool samples, with an average of 69,878 clean tags per sample after filtering (Supplementary Table S1). A total of 416 OTUs were obtained at a 97% similarity level among all samples based on the Silva database. The sequenced samples were taxonomically clustered into 9 phyla, 15 classes, 21 orders, 37 families, 97 genera, and 102 species (Supplementary Table S2).

The mouse gut microbiota harbored 21 bacterial phyla, with two dominant phyla (*Firmicutes* and *Bacteroidetes*) accounting for over 73.4% of the total relative abundance (Supplementary Figure S3A). The phylum *Firmicutes* was the most frequent taxon in the mouse gut at the phylum level, followed by *Bacteroidetes*, *Proteobacteria*, and *Epsilonbacteraeota*. We found that *Bacteroidia*, *Clostridia*, and *Bacilli* were the most abundant gut bacteria at the class level, with total average relative abundances of over 72.5% in the six groups (Supplementary Figure S3B). was the most prevalent abundant bacterium in all groups at the family level, followed by *Muribaculaceae*, *Rikenellaceae*, *Ruminococcaceae*, *Prevotellaceae*, and *Lactobacillaceae*. (Supplementary Figure S3C). At the genus level, uncultured bacteria of *Muribaculaceae* and *Alistipes* were the most prevalent gut microbiota detected in BS and NG group mice, while *Alloprevotella* and *Lactobacillus* were the most prevalent gut microbiota detected in SIBI and SI groups (Supplementary Figure S3D).

**FIGURE 2:**
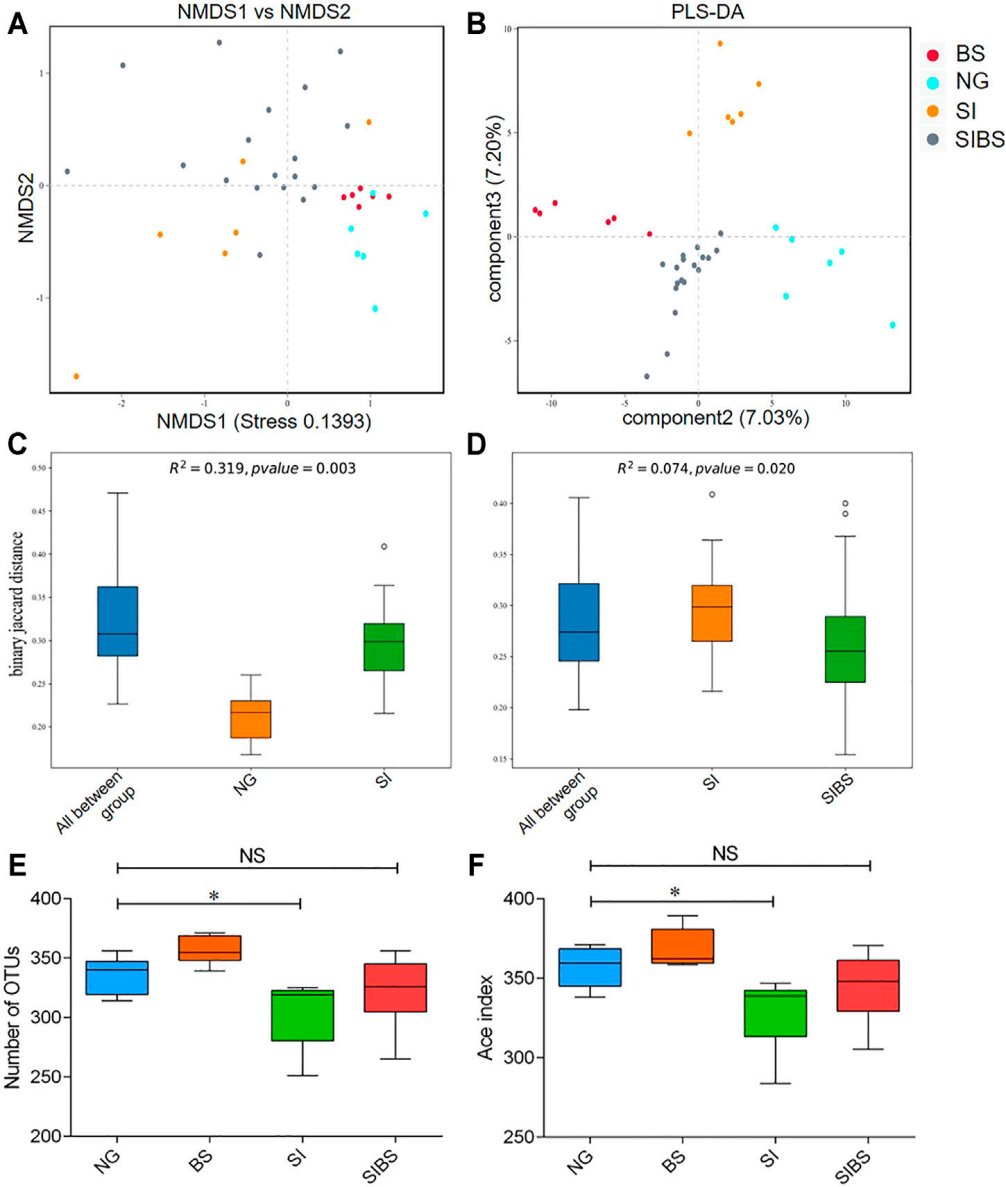
OA *B. subtilis* modulated community structures and alpha diversity of gut microbiota in *S. japonicum*–infected mice. **(A)** NMDS analysis. **(B)** PLS-DA analysis. **(C)** The difference in microbial communities between the ND and SI groups using PERMANOVA analysis. **(D)** The difference in microbial communities between the SI and SIBS groups using PERMANOVA analysis. **(E)** OTU number analysis. **(F**) ACE index analysis. NG: normal mice with PBS group. BS: normal mice with OA *B. subtilis* group. SI: *S. japonicum–*infected mice group. SIBS: *S. japonicum–*infected mice with OA *B. subtilis* group. * was considered statistically significant. NS: not statistically significant.

To evaluate bacterial community differences, we further analyzed the beta diversity of gut microbiota among groups. We found that the bacterial communities of normal mice with and without OA *B. subtilis* were similar (quantified by NMDS analysis) (Figure 2A). After *S. japonicum* infection, significant differences in bacterial community structures between infected mice and normal mice were found (quantified by NMDS and PLS-DA analysis, permanova: *p* < 0.05) (Figures 2A–C). Moreover, the community structures of the gut microbiota of *S. japonicum–*infected mice with OA *B. subtilis* were significantly different from those of infected mice (quantified by NMDS and PLS-DA analysis, permanova: *p* < 0.05) (Figures 2A,B,D). Our findings suggested that OA *B. subtilis* can significantly modulate the beta diversity of gut microbiota in *S. japonicum–*infected mice.

To evaluate how OA *B. subtilis* affects the diversity of the gut microbiota of *S. japonicum–*infected mice, we next analyzed the diversity of the gut microbiota in the intestinal contents. We found that *S. japonicum–*infected mice showed significantly lower diversities of gut microbiota than the control mice (Figures 2A,B). Interestingly, the alpha diversity of the gut microbiome of both infected and normal mice increased significantly after OA *B. subtilis* (Figures 2A,B). Our findings suggested that OA *B. subtilis* can modulate the alpha diversity of gut microbiota in *S. japonicum–*infected mice.

### Modulating the Composition of Gut Microbiota in *S. japonicum*–Infected Mice After OA *B. subtilis*

**FIGURE 3:**
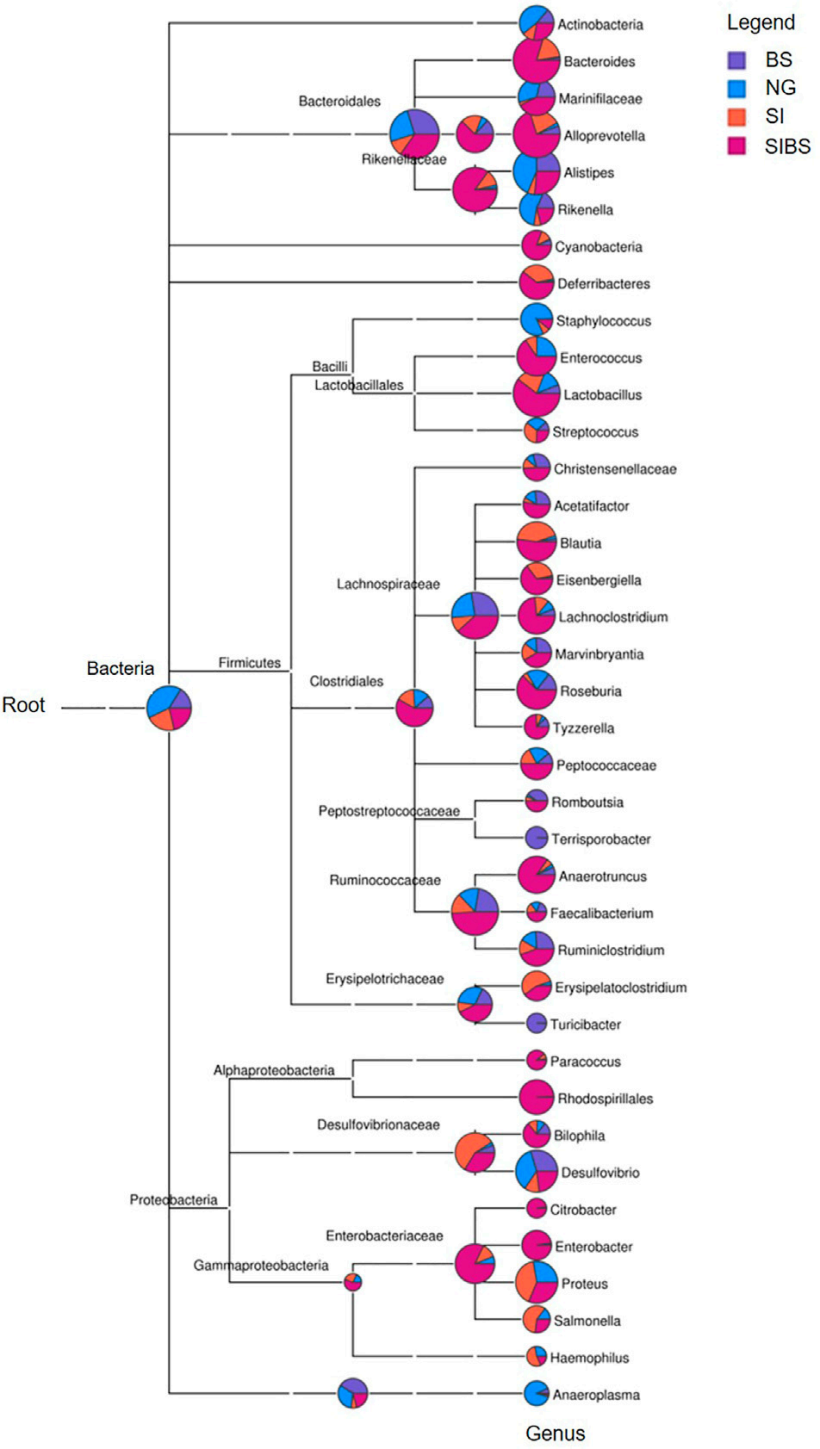
Phylogenetic diversity and taxonomical content of the gut microbiota sequences from all groups computed by MEGAN. In this figure, each circle represents a bacterial taxon in the NCBI taxonomy and is labeled by its name. The size of the circle represents the number of reads. NG: normal mice with PBS group. BS: normal mice with OA *B. subtilis* group. SI: *S. japonicum–*infected mice group. SIBS: *S. japonicum–*infected mice with OA *B. subtilis* group.

To further examine how OA *B. subtilis* affects the composition of the gut microbiota of *S. japonicum–*infected mice, we next performed taxonomic analysis using MEGAN. Differences in the top 38 bacterial taxa were noted among groups (Figure 3). SIBS mice had an overabundance of most bacterial genera, including *Bacteroides*, *Allporevotella*, *Enterococcus*, *Lactobacillus*, *Blautia*, *Lachnoclostridium*, *Anaerotruncus*, *Ruminiclostridium*, *Rhodospirillales*, and *Enterobacter* (Figure 3). Interestingly, most bacterial genera belonging to *Firmicutes*, which are considered beneficial bacteria, were overabundant in the SIBS population. Moreover, the ternary plot of microbial communities in different groups also revealed that the relative number of microbes belonging to the phylum *Firmicutes* was closer to the SIBS population than to the SI group (Supplementary Figure S4).

### Intestinal mRNA Expression Profiles of Mice Against Schistosome Infection by OA *B. subtilis*

**FIGURE 4:**
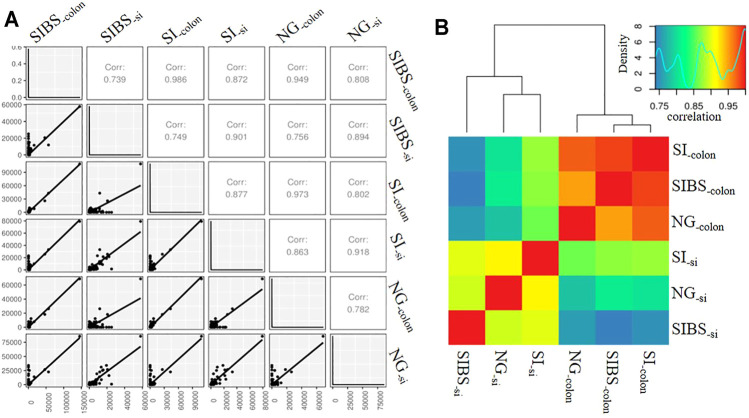
The correlation of intestinal mRNA expression profiles between groups. **(A)** Correlation shown by gene correlation. **(B)** Correlation shown by heatmap. NG: normal mice with PBS group. BS: normal mice with OA *B. subtilis* group. SI: *S. japonicum–*infected mice group. SIBS: *S. japonicum–*infected mice with OA *B. subtilis* group. “-colon” indicates colon tissue. “-si” indicates small intestine tissue.

Since OA *B. subtilis* significantly changed the gut microbiota composition and diversity of infected mice, we asked whether OA *B. subtilis* could also modulate the host response to schistosome infection. To this end, we performed small intestine and colon RNA-Seq in NG, SI, and SIBS mice after 8 weeks of infection. We found that the mRNA expression profiles between small intestine samples were strongly correlated (*r* ≥ 0.894), in addition to those between colon samples (Figure 4A). Meanwhile, there was also a strong correlation (*r* ≥ 0.739) between the small intestine and colon samples (Figure 4A). Similar results between sequenced samples are shown in the heatmap (Figure 4B). In addition, 275 of the total OTUs in the small intestine were commonly shared between the SI and SIBS groups (Supplementary Figure S5A). There were 330 OTUs in the small intestine uniquely identified in the NG versus (*vs*.) SIBS groups compared with the NG *v*s. SI groups. The shared and unique OTUs of expression profiles in the colon between groups are also displayed in the Venn diagram (Supplementary Figure S5B). These findings suggested that host immune system’s abilities between small intestine and colon to respond to intestinal and liver diseases are different.

**FIGURE 5:**
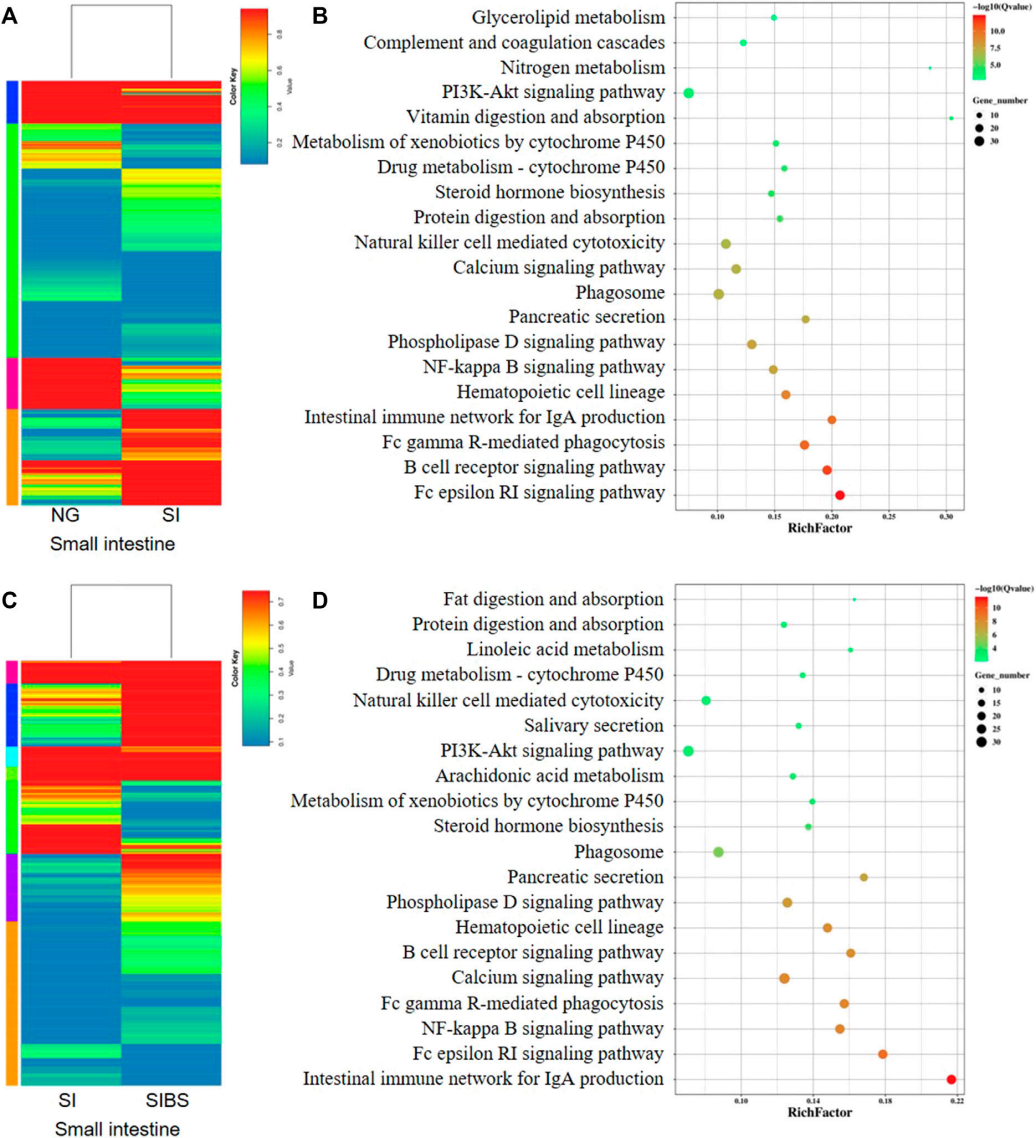
Comparison of DEGs in the small intestinal tissues were analyzed by KEGG enrichment and volcano diagram analysis. **(A,B)** Comparison of DGEs between the NG and SI groups. **(C,D)** Comparison of DGEs between the NG and SI groups. NG: normal mice with PBS group. SI: *S. japonicum–*infected mice group. SIBS: *S. japonicum–*infected mice with OA *B. subtilis* group. The size of each circle represents the number of genes.

The DEGs (based on fold changes) in small intestinal tissues between the NG and SI groups are shown as heatmaps in Figure 5A. Based on the assessments of the mRNA expression profiles of various genes in the small intestine of the NG *vs*. SI groups, we found that 514 genes were upregulated and 397 genes were downregulated (Supplementary Figure S6A; Supplementary Table S3). To identify the KEGG biological pathways enriched between the NG and SI groups, we revealed the top 10 most change enriched pathways, including the Fc epsilon RI signaling pathway, B cell receptor signaling pathway, Fc gamma R-mediated phagocytosis, intestinal immune network for IgA production, hematopoietic cell lineage, NF-kappa B signaling pathway, phospholipase D signaling pathway, pancreatic secretion, and phagosome (Figure 5B). Based on the KEGG classifications of DEGs, transport and catabolism, signal transduction, translation, lipid metabolism, and immune system ranked at the top of five KEGG categories, which included cellular processes, environmental information processing, genetic information processing, metabolism, and organismal systems (Supplementary Figure S7A). Based on the GO classification, these DEGs were classified into the cellular process, cell, and binding categories, ranking at the top of the biological process, cellular component, and molecular function classes, respectively (Supplementary Figure S8A). In addition, the DEGs (based on fold changes) in small intestinal tissues between the NG and SI groups are shown as heatmaps in Figure 5C. The comparative DEGs of small intestinal mRNA expression in profiles between the SI and SIBS groups revealed 541 upregulated and 348 downregulated genes (Supplementary Figure S6B; Supplementary Table S4). The KEGG pathway enrichment results between the SI and SIBS groups showed that the top 10 most prevalent pathways included the intestinal immune network for IgA production, Fc epsilon RI signaling pathway, NF-kappa B signaling pathway, Fc gamma R-mediated phagocytosis, calcium signaling pathway, B cell receptor signaling pathway, hematopoietic cell lineage, phospholipase D signaling pathway, pancreatic secretion, and phagosome (Figure 5D). The KEGG classification (Supplementary Figure S7B) and GO classification (Supplementary Figure S8B) are shown in the additional figures.

**FIGURE 6:**
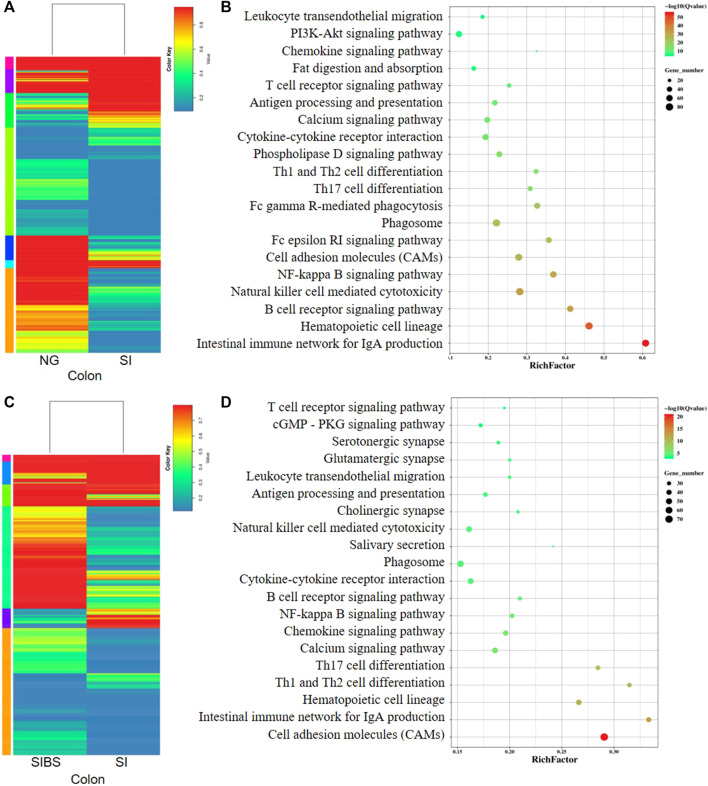
Comparison of DEGs in the colonal tissues were analyzed by KEGG enrichment analysis. **(A,B)** Comparison of DGEs between the NG and SI groups. **(C,D)** Comparison of DGEs between the SIBS and SI groups. NG: normal mice with PBS group. SI: *S. japonicum–*infected mice group. SIBS: *S. japonicum–*infected mice with OA *B. subtilis* group. The size of each circle represents the number of genes.

In addition, the comparative mRNA expression profiles in the colon of the NG vs. SI groups and SI *vs*. SIBS groups revealed that 1,693 genes (598 upregulated and 1,095 downregulated) (Supplementary Figure S6C; Supplementary Table S5) and 2,358 genes (1,783 upregulated and 575 downregulated) (Supplementary Figure S6D; Supplementary Table S6) were differentially expressed. They are shown as heatmaps in Figures 6A,C, respectively. The KEGG pathway enrichment results showed that the intestinal immune network for IgA production, hematopoietic cell lineage, B cell receptor signaling pathway, natural killer cell–mediated cytotoxicity, NF-kappa B signaling pathway, CAMs, Fc epsilon RI signaling pathway, phagosome, Fc gamma R-mediated phagocytosis, and Th17 cell differentiation were the top 10 most frequently enriched pathways in the colon between the NG and SI groups (Figure 6B). The KEGG classification (Supplementary Figure S7C) and GO classification (Supplementary Figure S8C) between the NG and SI groups are shown. In addition, CAMs, intestinal immune network for IgA production, hematopoietic cell lineage, Th1 and Th2 cell differentiation, Th17 cell differentiation, calcium signaling pathway, chemokine signaling pathway, NF-kappa B signaling pathway, B cell receptor signaling pathway, and phagosome were the 10 most change enriched pathways in the colon between the SIBS and SI groups (Figure 6D). Based on the DEGs between the SIBS and SI groups, the KEGG classification (Supplementary Figure S7D) and GO classification (Supplementary Figure S8D) were performed.

## Discussion and Conclusions

As one of the world’s most prevalent neglected tropical diseases, schistosomiasis affects public health in over 240 million people worldwide and results in approximately 70 million disability-adjusted life years lost annually (Colley and Bustinduy et al., 2014). Human blood flukes, including *S. japonicum* and *S. mansoni*, lay eggs in the portal venous system, and these eggs are subsequently trapped in the liver and intestine (Pearce and MacDonald, 2002). Schistosoma eggs could finally induce a variety of fibrotic diseases and lead to intestinal inflammation and gut microbiota dysbiosis (Gui et al., 2021; Holzscheiter et al., 2014; Hu et al., 2020). The probiotic bacterium *B. subtilis* could respond to hepatic and intestinal diseases and modulate the host gut microbiota (Lei et al., 2015; Rhayat et al., 2019; Wu et al., 2019). However, whether *B. subtilis* can protect mice against *S. japonicum* infection is unclear.

In this study, our findings indicated that OA *B. subtilis* significantly reduced intestinal fibrosis and hepatic fibrosis in *S. japonicum*–infected mice, as well as hepatic fibrosis. Fibrosis is the final pathological consequence of chronic inflammatory diseases, including schistosomiasis. Fibrotic diseases induced by *S. japonicum* infection affect many organs in mice and even cause mortality during the acute phase (Gunda et al., 2020; He et al., 2018; McManus et al., 2018). Praziquantel (PZQ), which has improved the prevention and control of schistosomiasis in endemic regions, is commonly used as an anti-schistosome drug (Colley and Bustinduy et al., 2014). However, until now, there have been no approved effective antifibrotic therapies (Wynn and Ramalingam, 2012). Although a mouse experiment showed that PZQ had specific antifibrotic effects (Liang et al., 2011). The clinical results showed that some patients with schistosome infection still developed to an advanced stage after treatment with PZQ. This development implied that PZQ could not significantly alleviate the fibrogranulomatous inflammation induced by schistosomal eggs (Colley et al., 2014). Therefore, in the clinic, PZQ plus liver protection by traditional Chinese medicine is usually used to prevent advanced schistosomiasis (Zhang and Xia, 2017; Yuan and Dai, 2021). Probiotics have been used as dietary supplements or medicinal supplements in humans, veterinary, and aquaculture (Duc et al., 2004; Cutting, 2011). Evidence suggests that *B. subtilis* supplementation attenuates hepatic injury (Wang et al., 2021). In addition, a previous study reported that *B. subtilis* contributes to the limitation of the intestinal inflammatory response and homeostasis in mice (Fujiya et al., 2007; Rhayat et al., 2019). Our study first revealed that OA *B. subtilis* alleviated liver and intestinal injury in mice infected by *S. japonicum*, implying that probiotic supplements may contribute to host biology during schistosomiasis.

The gut microbiota plays an important role in host health and immunity in all mammals (Browne et al., 2017). Dysbacteriosis of the gut microbiota can affect host health and biology and induce diseases such as cirrhosis (Bajaj et al., 2018; Kang et al., 2016) and inflammatory bowel disease (Lavelle and Sokol, 2020; Nishida et al., 2018). Therefore, homeostasis of the composition and diversity of gut microbiota is vital for biological aspects of the host (Eckburg et al., 2005; Liao et al., 2019). However, previous studies have demonstrated that schistosome infection can induce the loss and dysbiosis of gut microbiota in both mice (Cortes et al., 2020; Holzscheiter et al., 2014; Hu et al., 2020; Jenkins et al., 2018; Zhang B. et al., 2020) and humans (Gui et al., 2021; Kay et al., 2015). Our findings demonstrated that OA *B. subtilis* not only enhanced the alpha diversity but also remodeled the composition and community structures of the gut microbiome of *S. japonicum*–infected mice. Based on cohousing experiments, our previous study demonstrated that the gut microbiota plays a role in alleviating intestinal injury in mice during *S. japonicum* infection. A previous study demonstrated that the susceptibility to *Schistosoma mansoni* infection in mice partially depends on the composition of the host baseline microbiota, implying that the gut microbiota is associated with schistosome infection in rodent models (Cortes et al., 2020). These findings implied that the gut microbiota plays an important role in modulating intestinal and liver diseases induced by *S. japonicum* infection. For quite a long time, PZQ has been widely used for the treatment of schistosomiasis. However, PZQ acts against adult schistosome worms but plays a poor role in activity against deposited parasitic eggs (Cioli and Pica-Mattoccia, 2003; Colley et al., 2014). PZQ does not protect infected mice against fibrogranulomatous inflammation. Together, our study provides a new direction in which targeting the diversity of gut microbiota in *S. japonicum*–infected mice may attenuate hepatic and intestinal diseases.

Transcriptomics concerning schistosome–host interactions is an important tool for investigating new schistosome drug targets (Gobert and Jones, 2008). Previous studies on comprehensive transcriptomics have attempted to analyze host responses during the disease process, providing great insights into the dynamics of Th1/Th2 immune responses during schistosomiasis (Edungbola et al., 1982; Sandler et al., 2003). Nevertheless, the exact mechanisms of egg-induced fibrosis pathology remain largely unknown. To elucidate the role of OA *B. subtilis* in S. japonicum infection, we first analyzed the interaction between probiotics and the host using the transcriptome. The DEG profiles were different between the small intestine and colon in infected mice in our study. We also found a range of gut DEGs between infected mice with OA *B. subtilis* and those without OA *B. subtilis*. These DEGs were classified into KEGG pathways, including CAMs, intestinal immune network for IgA production, hematopoietic cell lineage, Fc epsilon RI signaling pathway, Th1 and Th2 cell differentiation, Th17 cell differentiation, calcium signaling pathway, Fc gamma R-mediated phagocytosis, chemokine signaling pathway, phospholipase D signaling pathway, NF-kappa B signaling pathway, B cell receptor signaling pathway, pancreatic secretion, and phagosome. DEGs in mouse DSS-induced inflammatory bowel disease were mainly classified into KEGG pathways, including the PPAR signaling pathway, influenza A, herpes simplex infection, synthesis and degradation of ketone bodies, measles, antigen processing and presentation, and ECM–receptor interaction (Wang et al., 2017). DEGs from transcriptomic profiles revealed that KEGG pathways involved in host responses to *Entamoeba histolytica* infection mainly included signal transduction, cytoskeletal rearrangement, proteasome activity, DNA repair factors, stress response, antimicrobial activity, vesicular trafficking, energy metabolism, virulence-related, detoxification pathway, transcriptional regulation, and translation and ribosome (Naiyer et al., 2019). These results suggested that host immune responses to different pathogens or nonpathogenic factors are remarkably different. In total, OA *B. subtilis* may alleviate intestinal injury by modulating gene expression profiles in mice during schistosomiasis.

In 2019, a total of 605,965 bovines were found in the schistosomiasis endemic areas of China, and 183,313 bovines underwent serological examinations with 1,176 positives detected (Zhang LJ. et al., 2020). In addition, previous studies have revealed that bovines play a major role in the transmission of S. japonicum in the lake and marshland regions in southern China (Gray et al., 2009; Guo et al., 2006). Mathematical modeling predicted that bovines are responsible for 75–90% of human blood flukes transmission (Gray et al., 2009; Guo et al., 2006). As we have known, parasitic infection can impact the body weight and immune system of the host. Moreover, *B. subtilis* is a common probiotic applied in agricultural and animal husbandry. We found that OA *B. subtilis* can increase the body weight of both *S. japonicum–*infected and normal mice and alleviate pathological injuries (Supplementary Figure S9). These findings implied that *B. subtilis* supplementation may be a potential control strategy for schistosomiasis in endemic regions.

Our findings demonstrated that OA *B. subtilis* attenuated intestinal and liver pathological injuries in *S. japonicum–*infected mice. We found that OA *B. subtilis* modulated the diversity and composition of gut microbiota in *S. japonicum*–infected mice. In addition, our results revealed that OA *B. subtilis* induced a range of DEG profiles in infected mice, which may be related to CAMs and intestinal immune network for IgA production. Our study may provide a potential complementary and therapeutic strategy for the treatment of human schistosomiasis.

## Data Availability

The datasets presented in this study can be found in online repositories. The names of the repository/repositories and accession number(s) can be found below: accession number SRR15682843–SRR15682848 (Transcriptomic data) and SRR15694234–SRR15694269 (16S rRNA gene sequences), respectively.

## Abbreviations

CAMs, cell adhesion molecules; cDNA, complementary DNA; DEGs, differentially expressed genes; dpi, days post-infection; DSS, dextran sulfate sodium; FDA, Food and Drug Administration; GO, Gene Ontology; HFD, high-fat diet; IOD, integrated optical density; KEGG, Kyoto Encyclopedia of Genes and Genomes; LB, lysogeny broth; NMDS, non-metric multidimensional scaling; OA, oral administration; OTU, operational taxonomic unit; PLS-DA, partial least squares discrimination analysis; PZQ, praziquantel.
