# Supplementary figures and images for: Bacillus subtilis Attenuates Hepatic and Intestinal Injuries and Modulates Gut Microbiota and Gene Expression Profiles in Mice Infected with Schistosoma japonicum
Source: Front Cell Dev Biol. 2021 Nov 16;9:766205. doi: 10.3389/fcell.2021.766205 (PMC8635066; doi:10.3389/fcell.2021.766205)

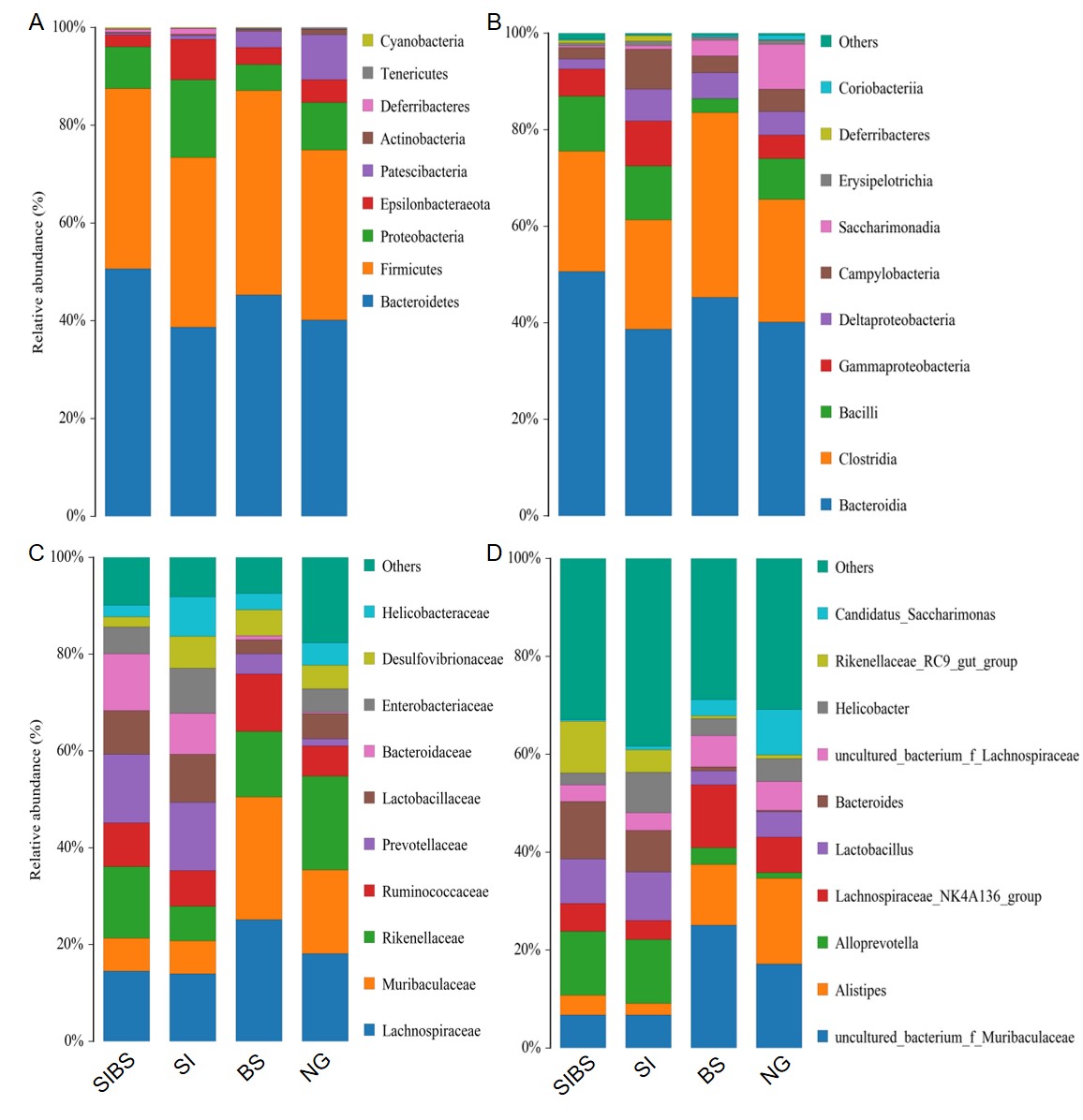

Supplement: Supplementary file 2 [file Image3.JPEG]

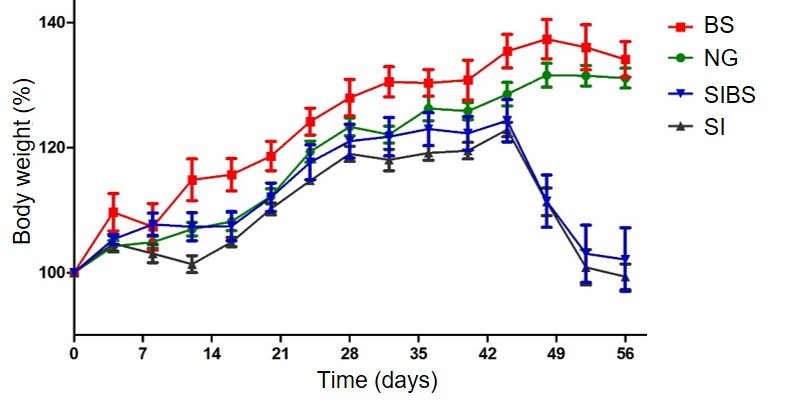

Supplement: Supplementary file 4 [file Image9.JPEG]

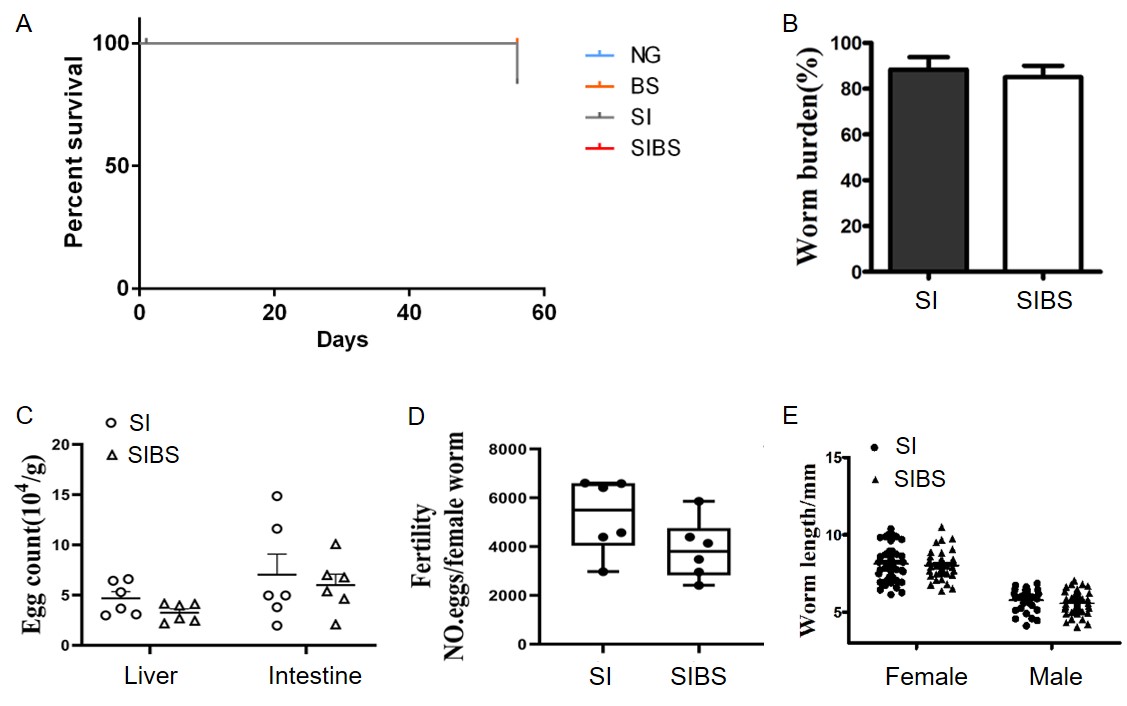

Supplement: Supplementary file 5 [file Image1.JPEG]

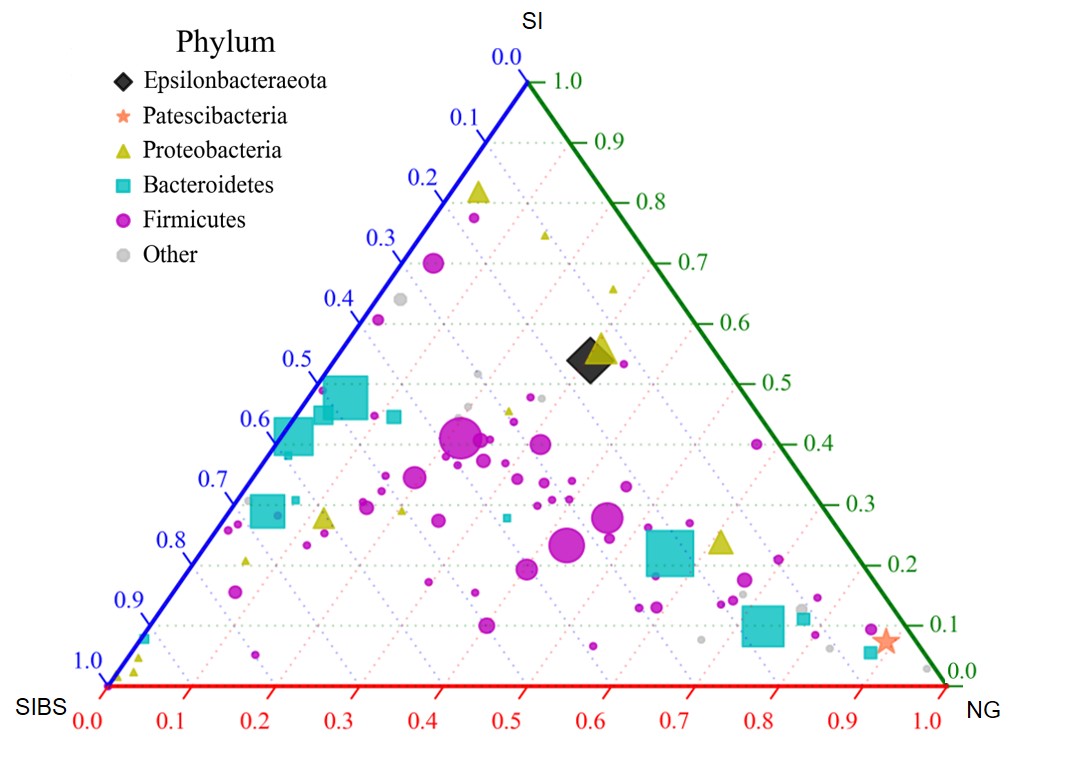

Supplement: Supplementary file 6 [file Image4.JPEG]

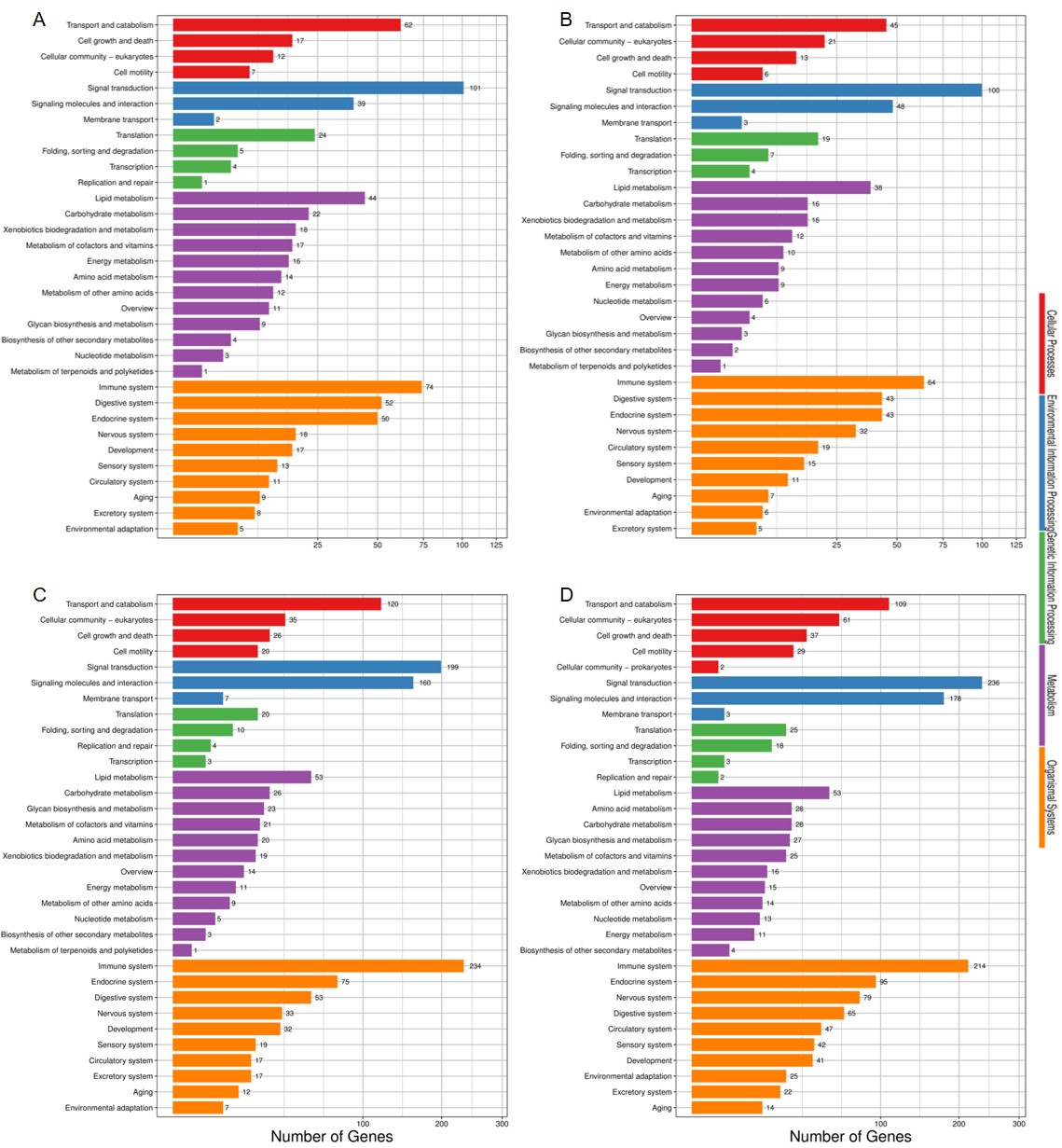

Supplement: Supplementary file 8 [file Image7.JPEG]

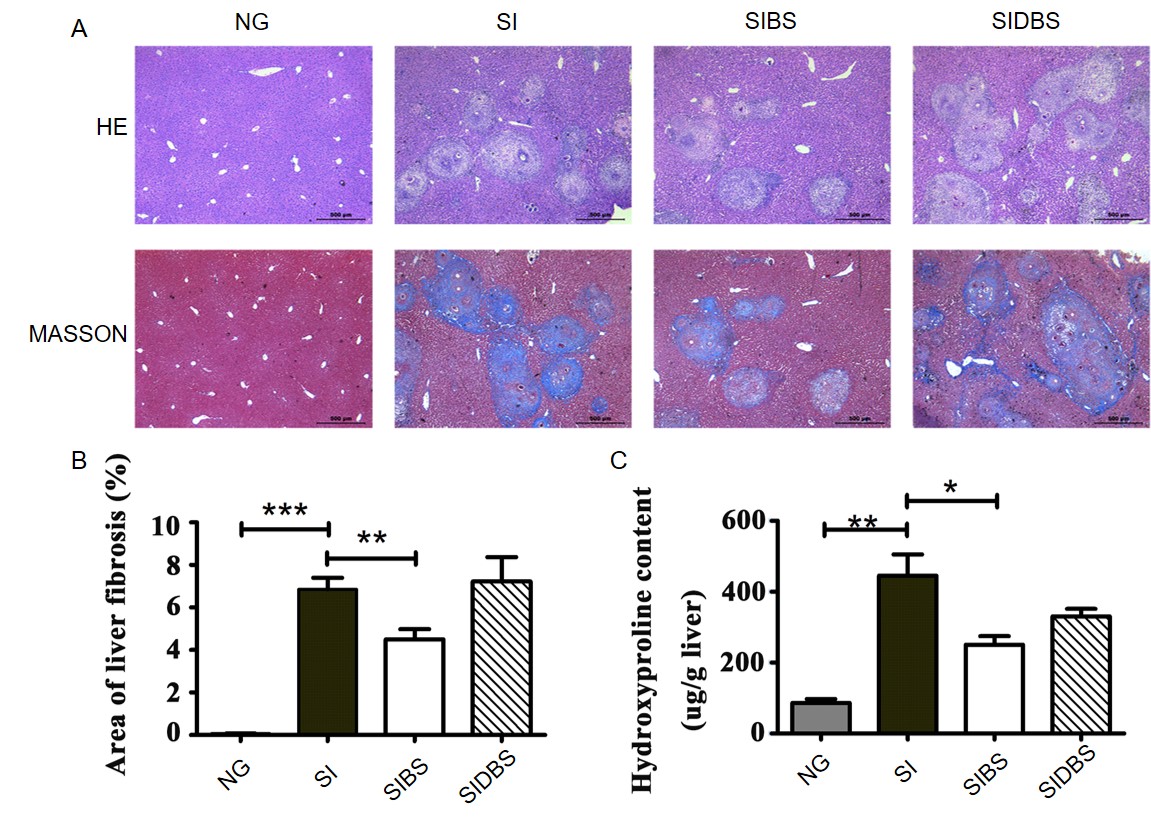

Supplement: Supplementary file 9 [file Image2.JPEG]

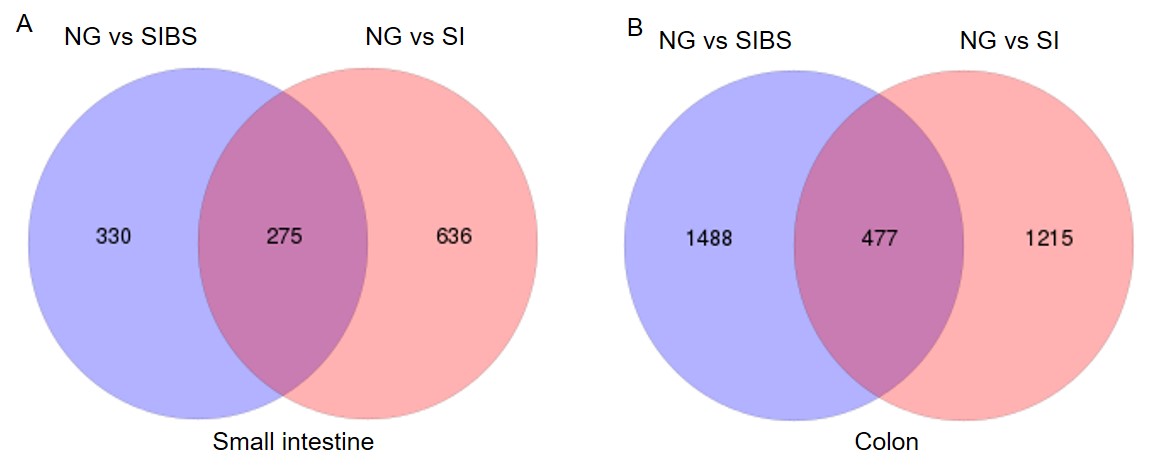

Supplement: Supplementary file 10 [file Image5.JPEG]

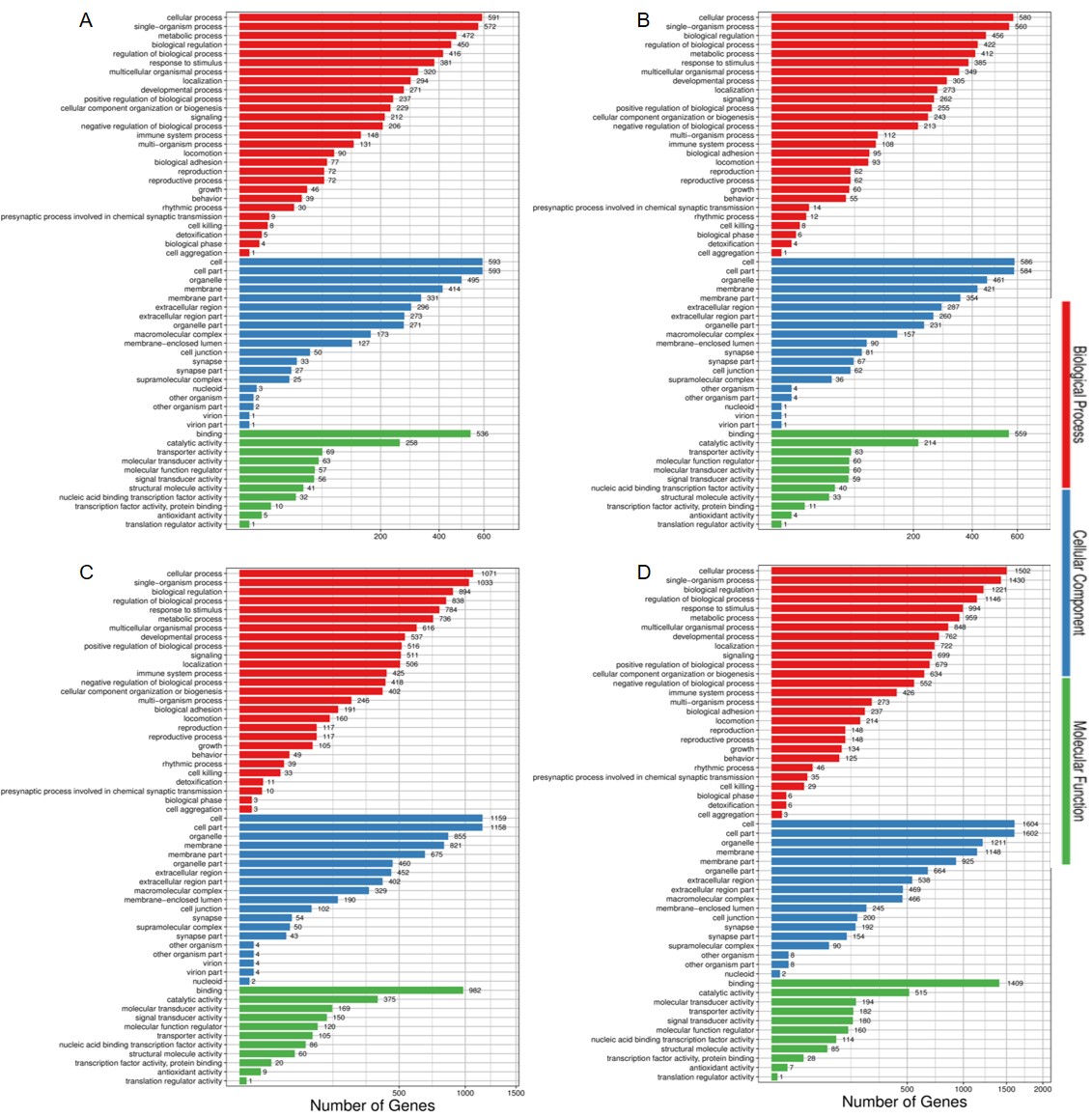

Supplement: Supplementary file 14 [file Image8.JPEG]

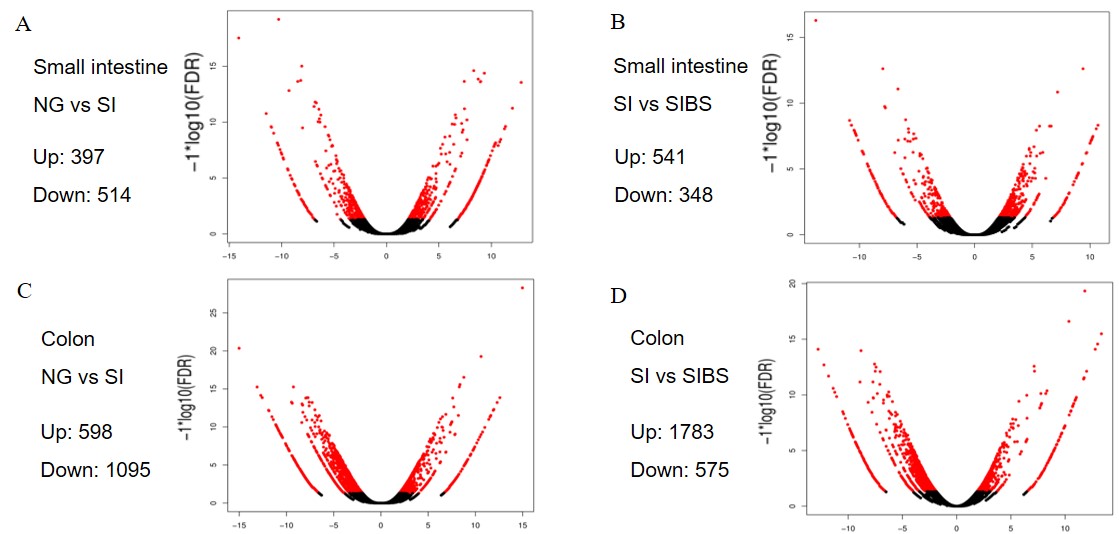

Supplement: Supplementary file 15 [file Image6.JPEG]
